# SETD7在肿瘤发生发展中的作用及机制的研究进展

**DOI:** 10.3779/j.issn.1009-3419.2023.106.02

**Published:** 2023-01-20

**Authors:** Limin CAO, Min WANG, Ke XU

**Affiliations:** 300052 天津，天津医科大学总医院，天津市肺癌研究所，天津市肺癌转移和肿瘤微环境重点实验室; Tianjin Key Laboratory of Lung Cancer Metastasis and Tumor Microenvironment, Tianjin Lung Cancer Institute, Tianjin Medical University General Hospital, Tianjin 300052, China

**Keywords:** SETD7, 甲基化, 组蛋白及非组蛋白底物, 肿瘤进展, SETD7, Methylation, Histone and non-histone protein, Tumor progression

## Abstract

肿瘤的发生是个复杂的过程，不仅取决于本身基因的突变或缺失，还受表观遗传调控的影响。近年来研究表明甲基化修饰在转录调控、异染色质的形成、X染色体失活、DNA损伤、肿瘤的发生发展等众多过程中均发挥着重要作用。含SET域赖氨酸甲基转移酶7（SET domain containing lysine methyltransferase 7, SETD7）是体内一种重要的赖氨酸甲基转移酶，可甲基化组蛋白，也可使非组蛋白甲基化。这种修饰调节方式一旦发生紊乱可直接导致细胞异常，从而引起多种疾病的发生。研究表明SETD7与多种肿瘤的发生发展具有相关性，但是有关SETD7甲基化底物位点及其在相应肿瘤中的调控机制并未完全阐明。本文将就SETD7对组蛋白及非组蛋白的甲基化修饰在肿瘤中发挥的作用及调控分子机制等方面的研究进展加以概述，以期为肿瘤的发病机制及诊疗研究提供新的治疗靶点。

## Abstract (en)

The occurence and development of tumors is a complicated process, which not only depends on the mutation or deletion of genes, but also is affected by epigenetic regulation. Accumulating evidences have shown that epigenetic modifications play fundamental roles in transcriptional regulation, heterochromatin formation, X chromosome inactivation, DNA damage response and tumor development. SET domain containing lysine methyltransferase 7 (SETD7) was initially identified as an important lysine methyltransferase, which methylated histone and non-histone proteins. These modifications play fundamental roles. Once this modification disorders, it can directly lead to cell abnormalities and cause many diseases. Studies have shown that SETD7 is related to the occurence and development of various tumors, but the methylation sites of SETD7 and its regulatory mechanism have not been fully elucidated. This article summarizes the research progress of the role of SETD7 on histone and non-histone methylation modification in tumors and the molecular mechanism, in order to provide new therapeutic targets for tumor pathogenesis and diagnosis.

SET结构域蛋白是具有蛋白赖氨酸甲基转移酶（protein lysine methyltransferases，PLMTs或PKMTs）活性的一类蛋白，其名称由最初报道表达该基序的三个基因，即su（var）3-9、enhancer of zeste和trithorax的首字母组合而来，主要通过修饰组蛋白H3K4、H3K9、H3K36、H3K27等位点及非组蛋白的赖氨酸残基位点，调控基因的表达^[1]^。该蛋白表达于所有的真核生物中，由约130个氨基酸组成。早在2000年，作为组蛋白赖氨酸甲基化的里程碑，第一个组蛋白甲基化转移酶SUV39H1和其酵母同源基因Clr4被报道^[2]^。Yu等^[3]^的研究表明SUV39H1可抑制前列腺癌的转移。而其次被报道的甲基转移酶KMT1C在白血病、前列腺癌、肝癌、肺癌和乳腺癌等多种疾病中均异常高表达^[4⇓⇓-7]^。随着研究的不断深入，目前已报道SET家族共包含SUV3-9、SET1、SET2、SMYD、EZ、SUV4-20、RIZ、SET7/9及SET8九大类，这些酶均以S-腺苷甲硫氨酸（S-adenosyl-L-methionine, SAM）作为甲基供体，调控SAM上的甲基转移到相应的组蛋白或非组蛋白底物赖氨酸或精氨酸上，从而影响基因的表达^[8,9]^。

已有研究^[1]^表明SET家族与肿瘤以及多种疾病的发生密切相关，甚至同一种酶的不同作用位点在不同的肿瘤中发挥着不同的生物学效应。组蛋白3的第9位、第27位及组蛋白4的第20位上发生的赖氨酸甲基化修饰与转录抑制相关，而组蛋白3第4位、第36位、第79位上发生的赖氨酸甲基化修饰与转录激活相关。Zeste基因增强子同源物2（enhancer of Zeste homolog 2, EZH2）可调节位点组蛋白3上的第27位赖氨酸（histone H3 lysine 27, H3K27）的三甲基化。EZH2在结肠癌、胃癌、膀胱癌、黑色素瘤、淋巴瘤等多种肿瘤中异常表达，研究^[10⇓⇓-13]^显示其表达增高与乳腺癌、前列腺癌等肿瘤的转移相关。核受体结合SET结构域蛋白1（nuclear receptor-binding SET domain protein 1, NSD1）催化H3K36双甲基化，促进骨髓瘤的发生^[14]^。Suv39h1（variegation 3-9 homolog1）主要介导H3K9的三甲基化，其异常高表达与前列腺癌的发生密切相关^[15]^。甲基转移酶（mixed lineage leukemia gene 1, MLL1）可双甲基化H3K4参与白血病的发生^[16]^。SMYD3（SET and MYND domain containing protein 3）在乳腺癌、肝癌、结肠癌、胃癌等多种类型的肿瘤中高表达，与转录激活相关^[17]^。含SET域赖氨酸甲基转移酶7（SET domain containing lysine methyltransferase 7, SETD7）可调节H3K4甲基化，研究^[18,19]^显示SETD7可参与肝癌、乳腺癌、胃癌、白血病等多种恶性肿瘤的发生发展。以上研究均证明SET家族蛋白的修饰异常与肿瘤具有密切相关性。随着研究的不断深入，发现了一些靶向组蛋白甲基转移酶的分子抑制剂，其中BIX01294作为第一个报道的组蛋白赖氨酸甲基转移酶抑制剂表现出良好的抗肿瘤效果，为肿瘤的治疗提供了新思路^[20,21]^。Cyproheptadin被报道可以作为SETD7的小分子抑制剂，通过降低内质网应激相关蛋白CHOP和H3K4me1的表达来预防高血糖诱导的肾纤维化及炎症反应^[22]^。正因如此，SET家族蛋白受到越来越多的关注。本文主要对SETD7修饰的底物及与肿瘤的相关性及调控分子机制等方面的研究进展加以概述，为肿瘤的诊疗提供新的思路及靶点。

## 1 SETD7的发现及结构

SETD7是PLMTs家族中的一员，最早于HeLa细胞的细胞核中提取获得，也有报道将其称为KMT7、SET7/9、SET7或SET9，基因位于人的第4号染色体（4q28）上，蛋白产物均含有保守的SET结构域，包括N端和C端，相对分子质量约为50 kDa。SETD7介导的甲基化可调控蛋白质和蛋白质之间以及蛋白质和核酸之间的相互作用。其中N端主要功能是维持酶自身结构的稳定性，C端主要发挥催化的作用，结合辅助因子SAM提供的甲基，将甲基转移到底物赖氨酸或者精氨酸相应的修饰位点上，最初被认为是H3K4的特异性赖氨酸甲基转移酶。随着研究的深入，发现SETD7还可甲基化p53、E2F1、TATA框结合蛋白结合因子10[TATA box binding protein (TBP)-associated factor, TAF10]、雌激素受体α（estrogen receptor α, ERα）、信号转导及转录激活蛋白3（signal transducer and activator of transcription 3, STAT3）、Yap等非组蛋白，影响众多基因的功能。和其他含有SET结构域的PLMTs一样，SETD7也具有独特的由β折叠环绕成绳结样。早期的一些研究^[23⇓-25]^结果表明SETD7可激活肌肉分化关键基因肌细胞生成素（myogenin, MyoG）的转录，促进肌细胞的分化。

## 2 SETD7的甲基化修饰功能

### 2.1 SETD7对组蛋白的甲基化修饰作用

组蛋白的甲基化修饰是一个动态的、可逆的过程。核小体是由约150个碱基组成的DNA和组蛋白（H2A, H2B, H3, H4）共同组成。其每个组蛋白都有N端拖尾伸出核小体之外，这些拖尾可发生磷酸化、甲基化、泛素化、乙酰化等多种转录后修饰。这些修饰并不改变碱基序列，但是可以使基因的表达发生变化且可遗传，即表观遗传修饰^[26]^。SETD7于2002年被发现，其对染色体的维持起着重要的作用^[27]^。Tao等^[23]^发现，SETD7可催化H3K4发生单甲基化修饰，促进成肌细胞分化成肌管，从而促进骨骼的发育。Sankrityayan等^[22]^发现SETD7可单甲基化组蛋白H3K4，从而预防高糖导致的肾纤维化及炎症反应。Fujimaki等^[28]^发现，SETD7甲基化H3K4后激活胰腺细胞中的一氧化氮合成酶2（nitric-oxide synthase 2, NSO2）转录，促进炎性细胞因子的传导。Tuano等^[29]^发现，SETD7可催化H3K4甲基化，其可激活小鼠胚胎干细胞中分化基因的转录，促进胚胎干细胞分化。在急性淋巴母细胞性白血病中，SETD7能调节H3K4甲基化，进一步激活基因的表达^[30]^。以上研究表明SETD7可通过修饰组蛋白参与细胞的分化，影响细胞周期及蛋白表达等过程。

### 2.2 SETD7对非组蛋白修饰作用

除了组蛋白，SETD7还可修饰一些肿瘤抑制因子、膜上相关受体、转录因子等非组蛋白，其作用与转录激活、蛋白稳定性具有相关性。现已报道的甲基化底物有Gli3、p53、E2F1、TAF10、ERα、DNA甲基转移酶1（DNA methyltransferase 1, DNMT1）、RelA、STAT3、YY1和Yap等，具体非组蛋白底物及在肿瘤中的作用详见表1^[31⇓⇓⇓⇓⇓⇓⇓⇓⇓⇓⇓⇓⇓⇓⇓⇓⇓⇓⇓⇓⇓⇓-54]^。Fu等^[53]^发现，SETD7可甲基化转录因子Gli3上的K436、K595位点，从而激活Sonic Hedgehog信号通路，在哺乳动物的发育中发挥重要作用。SETD7可甲基化成视网膜母细胞瘤蛋白（retinoblastoma protein, pRb）的K873和K810位点，阻止细胞周期进入S期，从而促使细胞分化影响细胞衰老^[31]^。SETD7能使p53的K372、K369位点发生单甲基化，甲基化后的p53稳定性增加，可进一步增强对靶基因的活化作用。有研究^[32]^发现，SETD7甲基化p53时需先结合乙酰转移酶Tip60，进行乙酰化修饰，其在DNA损伤应答中发挥重要的作用。SETD7可催化p65上的K314、K315位点甲基化后直接激活核因子-κB（nuclear factor kappa B, NF-κB）信号通路，甲基化后的p65稳定性减弱，泛素化修饰增强，p65和DNA结合的蛋白酶体降解^[34]^。SETD7也可催化p65上的K37发生甲基化，甲基化后的p65和炎症基因[肿瘤坏死因子α（tumor necrosis factor α, TNFα）、单核细胞趋化蛋白-1（macrophage chemoattractant protein 1, MCP-1）、白细胞介素-8（interleukin 8, IL-8）]启动子结合的能力变强^[35]^。SETD7可甲基化雌激素受体（estrogen receptor α, ERα）肽链第302位赖氨酸，其蛋白稳定性增加从而增强雌激素对靶基因的激活^[36]^。DNA损伤后SETD7催化其衍生因子E2F1的K185位点受到抑制，促进E2F1的乙酰化和磷酸化过程，抑制凋亡^[54⇓-56]^。SETD7可将Flap核酸内切酶1（Flap endonuclease 1, FEN1）的K377位点进行单甲基化，在DNA损伤应答中发挥重要的作用^[37]^。也可单甲基化DNA结合转录因子YY1（Yin Yang 1）上的K247位点，从而影响其结合DNA能力进而影响基因组的稳定性及细胞增殖能力^[38]^。SETD7可以甲基化TAF10的折叠结构域第2个环处的第189位赖氨酸，该修饰可以增强TAF10对RNA聚合酶II的亲和力，增强转录^[57]^。Han等^[58]^报道SETD7在AR连接受体的DNA结合域和配体结合域的铰链域中的Lys-362处甲基化。SETD7也被报道可以在Lys-51处单甲基化TAT，共激活人类免疫缺陷病毒（human immunodeficiency virus, HIV）基因的表达，在HIV发病机制中发挥着一定的作用^[25]^。随着研究的不断深入，SETD7甲基化非组蛋白的作用受到越来越多的关注。

**表1:** SETD7的非组蛋白底物、甲基化位点及在肿瘤中的作用

Non-histone substrates	Lysine residues	Role of SETD7 in tumors
pRb	K873, K810	Inhibit cell growth in osteosarcoma^[31]^
p53	K372, K369	Cell cycle arrest induced by activation p21/WAF/CIP pathway in osteosarcoma^[32]^ Induce colorectal cancer cells apoptosis^[33]^
p65	K314, K315, K37	Inhibit activating NF-κB in A549^[34]^ Activate downstream pathways in HeLa^[35]^
ERα	K302	Increase sensitivity of ERα to estrogens in breast cancer, SETD7 as an indicator of poor prognosis in breast cancer^[36]^
FEN1	K377	Participate in the stress response of cells^[37]^
YY1	K247	Activate transcription^[38]^
AR	K189	Enhances AR activity, tumor promoter in prostate cancer^[39]^
TAT	K51	Some roles in HIV^[40]^
Sam68	K208	Impair Sam68 nuclear activity, tumor promoter in colorectal cancer^[39]^
β-catenin	K180	Tumor promoter in HeLa cells^[41]^
eL42	K53, K80, K100	Protein stability^[42]^
YAP	K494	Increase tumorigenicity of overexpression SETD7^[43]^
FXR	K206	Activate transcription^[44]^
PCAF	K89	Unknown
SUV39H1	K105, K123	Promote cell proliferation in Saos-2^[45]^
HIF-1α	K32	Negatively regulates HIF-α transcriptional activity^[46]^
FOXO3	K270, K271	Unknown
UHRF1	K385	Methylation of UHRF1 by SETD7 is essential for DNA double-strand break repair^ [47]^
DNMT1	K142, K1096	Induce EMT in breast cancer cells^[48]^
SIRT1	K233, K235, K236, K238	Involved in DNA damage in prostate cancer^[49]^
RUNX2	H3K4	Promote proliferation, migration and invasion of breast cancer cells^[50]^
Gli-1	Unknown	Inhibit oncogenic activities through regulation of Gli-1 expression in breast cancer^ [51]^
ARTD1	K508	Involved in cellular oxidative stress response^[52]^
Gli3	K595, K436	Promote the growth and metastasis of lung cancer cells^[53]^
E2F1	K185	Promotes hepatocellular carcinoma progression^[54]^

HIV: human immunodeficiency virus; EMT: epithelial mesenchymal transition; pRb: retinoblastoma protein; ERα: estrogen receptor α; YY1: Yin Yang 1; HIF-α: hypoxia-inducible factor-alpha; SETD7: SET domain containing lysine methyltransferase 7; NF-κB: nuclear factor kappa B; DNMT1: DNA methyltransferase 1.

## 3 SETD7与肿瘤的相关性及其调控机制

赖氨酸甲基转移酶SETD7除了上述作用，还与乳腺癌、胃癌、肝癌、肺癌等多种恶性肿瘤密切相关，SETD7在肿瘤的诊断和治疗中表现出一定的应用前景。

乳腺癌作为威胁妇女健康的重要恶性肿瘤之一，在我国表现出明显的年龄和地区差异。然而SETD7与转录因子如何相互作用来调节乳腺肿瘤的发生仍不清楚。Zhang等^[59]^发现，SETD7在80例乳腺癌患者中明显上调，并与血管内皮生长因子（vascular endothelial growth factor, VEGF）的表达及微血管数量呈正相关，SETD7可作为独立的乳腺癌预后不良的指标；体外实验研究表明在人乳腺癌细胞系MCF7、ZR-75-1、MDA-MB-231中敲低SETD7可有效抑制细胞的增殖、迁移及侵袭，体内实验结果显示敲低SETD7基因后明显抑制肿瘤的生长。Song等^[51]^发现，乳腺癌细胞中SETD7表达降低会促进细胞的增殖、迁移及侵袭，反之可抑制细胞增殖、迁移及侵袭。Montenegro等^[60]^发现，在乳腺癌细胞MDA-MB-231中过表达SETD7可抑制细胞的增殖迁移，敲低该基因后便增强了细胞的迁移侵袭能力。Si等^[50]^利用人类蛋白质图谱和基因表达综合（Gene Expression Omnibus, GEO）数据库，分析了SET7/SET9的表达，在人乳腺癌细胞系MCF-7和MDA-MB-231构建异种移植肿瘤模型，利用质谱、免疫共沉淀、GST pull-down和泛素化测定进行机制研究，结果表明在乳腺癌中SETD7的表达与患者生存期负相关，体内研究和体外研究显示SETD7可以通过激活RUNX2促进细胞的迁移和侵袭能力，SETD7可以与蛋白TRIM21相互作用参与泛素化。不同研究者的报道结果显示SETD7在乳腺癌的发生发展中发挥复杂的生物学效应。

肺癌的死亡率居于第一位，其转移是导致患者治疗失败和死亡的主要原因。Daks等^[61]^发现，利用CRISPR/Cas9敲低肺腺癌细胞株A549以及非小细胞肺癌（non-small cell lung cancer, NSCLC）细胞株H1299中的SETD7，结果显示细胞的增殖速度增加，分子机制表明敲除SETD7细胞周期蛋白Cyclin A2和Cyclin D1表达上调，E-cadherin表达下调，其产物对细胞间的相互作用至关重要，从而导致细胞的迁移能力增强，影响了细胞对阿霉素的敏感性；同时发现SETD7敲低后显著提高了肺癌细胞的线粒体膜电位（mitochondrial membrane potential, MMP）、糖酵解、呼吸和增殖率，显示SETD7可能参与肺癌细胞的能量代谢。本课题组在细胞实验中同样发现SETD7在肺癌患者组织中的表达低于配对的癌旁组织，在NSCLC细胞系H1299、A549中敲低SETD7可以促进肺癌细胞的迁移和侵袭，转移相关基因基质金属蛋白酶2（matrix metalloproteinase 2, MMP2）、VEGF表达增加^[62]^。Lezina等^[63]^利用short hairpin RNA干扰p53缺陷但是表达E2F1的NSCLC细胞H1299中SETD7的表达，结果表明可诱导细胞凋亡，流式结果显示细胞停滞在G_1_期/S期，进一步机制研究表明SETD7可以修饰E2F1进一步影响下游基因TP73的表达，E2F1在肺癌中发挥致癌的作用，SETD7同时可以负调控TP73，临床病例数据分析显示高表达TP73组生存较差。Gu等^[30]^发现，SETD7在肺癌细胞系H1299及A549中的表达水平高于正常细胞HEK293细胞，H1299细胞过表达SETD7后，抑制细胞凋亡，促进细胞增殖，转录子E2F1表达下调，敲低SETD7后促进细胞凋亡，E2F1表达上调。以上所有的研究结果表明甲基转移酶SETD7在肺肿瘤细胞中表现出矛盾的生物学效应，有可能由细胞的状态或环境及修饰位点的不同造成。

胃癌目前是全球发病率第五位的恶性肿瘤，在肿瘤致死率中居第三位。Akiyama等^[64]^研究了376例原发性胃癌患者，统计学结果显示其中129例伴随着SETD7缺失或低表达，与高表达组相比，缺失或低表达SETD7组表现出较低的生存率、临床高侵袭性以及预后不良。实验结果显示胃癌细胞MKN74、MKN45、AGS及KATO-III中敲低SETD7会引起SREK1IP1、PGC和CCDC28B基因表达下调，转移相关基因MMP1、MMP7、MMP9明显上调，并且明显促进细胞的增殖、迁移和侵袭能力；机制研究表明SETD7在SREK1IP1转录起始位点上游4 kb-6 kb处和启动子PGC和CDC28B结合，单甲基化位点H3K4，该研究表明SETD7的缺失或低表达促进胃癌的发生发展。

肝癌是最常见的恶性肿瘤之一，在我国恶性肿瘤死亡率中排名第二。Gu等^[54]^发现，与正常肝细胞LO2相比，肝癌细胞系SMMC-7721、Bel-7404、Huh7、HepG2、QGY-7703、HCC-0010中SETD7表达均明显升高，肝癌细胞系SMMC-7721中SETD7被敲低后，促进细胞的凋亡，其迁移能力明显降低，而HepG2及Bel-7404过表达该基因后，凋亡率降低，迁移能力增强。而Chen等^[65]^发现，肝癌临床标本癌组织中SETD7表达水平明显高于癌旁组织，显示SETD7在癌组织中的高表达与患者转移、复发、肿瘤的大小、预后不良密切相关。肝癌细胞系HepG2中过表达SETD7后促进细胞的增殖，肝癌细胞系SMMC-7721中敲低SETD7后通过调节细胞周期抑制细胞的增殖。以上研究显示SETD7在肝癌的发生和发展中发挥出了复杂的生物学效应。

骨肉瘤（osteosarcoma, OS）是一种常见的原发性骨恶性肿瘤，在儿童和青年人中具有侵袭性和远端转移性。研究^[66]^表明骨肉瘤细胞系Saos-2中敲除SETD7后促进细胞的增殖，并引起Sirtuin1蛋白水平上调，机制表明SETD7可甲基化Suv39h1，使基因组不稳定，促进细胞增殖和分裂。Lezina等^[63]^发现p53阳性的U2OS细胞中缺乏SETD7的细胞其细胞周期停滞，利用染色质免疫共沉淀发现SETD7可甲基化转录因子E2F1，CCNE1的启动子处H3K9Me3升高从而影响细胞周期蛋白E的表达，机制研究表明p21/WAF/CIP在DNA损伤中发挥了重要的作用。

卵巢癌的死亡率在女性生殖系统的恶性肿瘤中位列第一。已有研究^[67]^显示靶向SETD7的miR-153可抑制卵巢癌细胞的增殖和迁移，研究发现抑制SETD7表达后，p53乙酰化水平升高，促进卵巢癌细胞的凋亡。急性髓性白血病（acute myeloid leukemia, AML）是异质性的以恶性胚细胞在患者骨髓积累为特征的一组血液恶性肿瘤，Gu等^[30]^发现，在8例正常人骨髓血样中均检测到SETD7呈现高表达，而35例患者其中18例表现出低表达甚至缺失表达，5株白血病细胞系NB4、HEL、HL-60、THP-1、KG-1均检测到高表达SETD7，KG-1a未检测到表达，在细胞KG-1a中过表达SETD7后促进细胞的凋亡，细胞中敲除KG-1基因后抑制细胞凋亡。在结直肠癌中，甲基转移酶SETD7可修饰Sam68，位点为K208，生物信息学分析^[41]^显示患者中异常高表达SETD7与Sam68则生存率更高。甲基转移酶SETD7在胶质瘤细胞中低表达，机制表明SETD7可介导DRAIC启动子上的H3K4me3富集^[68]^，通过靶向miR-18a-3p调节胶质瘤细胞的生长和转移。

## 4 展望

不同研究者在人乳腺癌、人胃癌、肺癌、肝癌、人卵巢癌及白血病等恶性肿瘤中的研究结果表明甲基转移酶SETD7在肿瘤细胞中的调控作用具有双重性，在不同组织类型肿瘤细胞中呈现不同的生物学效应。进一步表明其修饰底物和修饰位点的复杂性。这种多样性的作用是由于SETD7的作用机制复杂，不单对组蛋白甲基化，还可以对非组蛋白甲基化，而针对后者的研究目前还处于探索阶段，需更加深入系统地展开研究。同时，还需在不同类型肿瘤的细胞系及临床样本中开展更深入的研究，使SETD7在肿瘤发生及转移过程中的作用更加清晰，为以SETD7为靶标的肿瘤治疗和抗癌药物研发提供理论和实验依据。
